# Anion-Exchange Membrane Oxygen Separator

**DOI:** 10.1021/acsorginorgau.4c00052

**Published:** 2024-08-29

**Authors:** Maisa Faour, Karam Yassin, Dario R. Dekel

**Affiliations:** a The Wolfson Department of Chemical Engineering, Technion − Israel Institute of Technology, Haifa 3200003, Israel; b The Nancy & Stephen Grand Technion Energy Program (GTEP), Technion − Israel Institute of Technology, Haifa 3200003, Israel; c The Stewart and Lynda Resnick Sustainability Center for Catalysis, Technion − Israel Institute of Technology, Haifa 3200003, Israel

**Keywords:** Anion exchange membrane, Electrochemical oxygen separation, Oxygen purification, Cell performance, Alkaline oxygen reduction reaction, Alkaline oxygen evolution reaction, Modeling

## Abstract

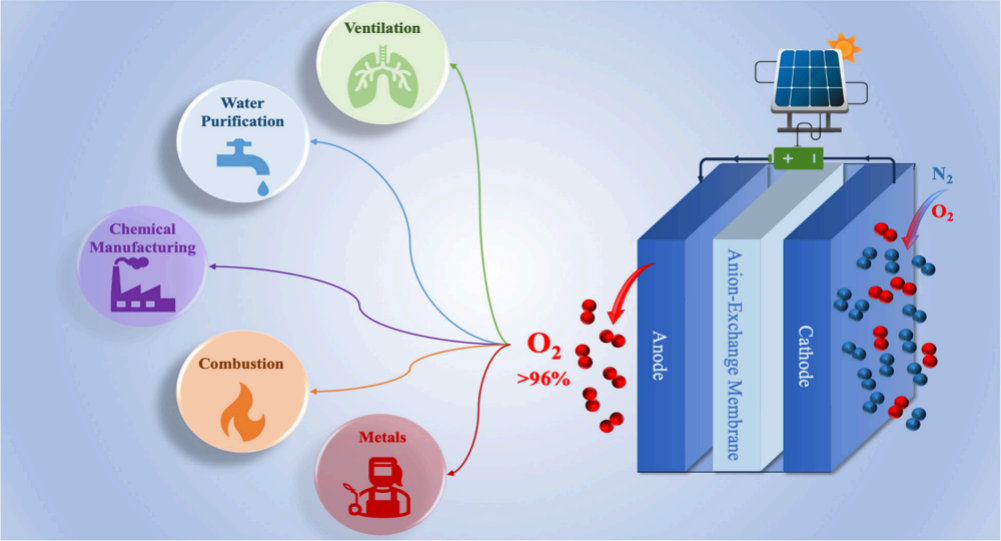

Anion-exchange membranes (AEMs), known for enabling the high conductivity of hydroxide anions through dense polymeric structures, are pivotal components in fuel cells, electrolyzers, and other important electrochemical systems. This paper unveils an unprecedented utilization of AEMs in an electrochemical oxygen separation process, a new technology able to generate enriched oxygen from an O_2_/N_2_ mixture using a small voltage input. We demonstrate a first-of-its-kind AEM-based electrochemical device that operates under mild conditions, is free of liquid electrolytes or sweep gases, and produces oxygen of over 96% purity. Additionally, we develop and apply a one-dimensional time-dependent and isothermal model, which accurately captures the unique operational dynamics of our device, demonstrates good agreement with the experimental data, and allows us to explore the device’s potential capabilities. This novel technology has far-reaching applications in many industrial processes, medical oxygen therapy, and other diverse fields while reducing operational complexity and environmental impact, thereby paving the way for sustainable on-site oxygen generation.

## 1 Introduction

As the global demand for high-purity oxygen continues to rise across many industrial sectors, the development of efficient oxygen separation technologies is gaining more traction.^1−3^ Advancements in electrochemical science have led to the emergence of modern technologies, which leverage principles of electrochemistry to selectively separate desired species from gas mixtures.^3−6^ Solid oxide technologies are the front runners in electrochemical oxygen separation, offering high oxygen-ion selectivity and a variety of conductive ceramic materials.^7−9^ While the high working temperature of these devices (>600 °C) is necessary to achieve a high ionic selectivity, it can greatly impair the integrity and stability of the cells due to thermal expansion and degradation of their materials, driving the technology further away from industrial development and necessitating the research and development of other technologies.^10−12^

Anion-exchange membranes (AEMs) play a crucial role in fuel cells, electrolyzers, and other electrochemical devices, by facilitating the selective transport of hydroxide anions while blocking the transport of other undesired cationic species.^13−17^ Researchers have made strides in tailoring the chemical structure of AEMs to improve their hydroxide conductivity and alkaline stability, addressing longstanding challenges associated with membrane degradation and performance loss over time.^18−24^ Furthermore, the alkaline conditions of AEM-based devices enable the use of earth-abundant, precious-metal-free catalysts such as nickel, iron, cobalt, and even metal-free heteroatom-doped carbons, offering a cost-effective alternative to platinum-group metal (PGM) catalysts.^24−29^ This shift toward PGM-free catalysts aligns with the efforts to make electrochemical technologies more economically viable and environmentally friendly.

In the realm of electrochemical oxygen separation technologies in alkaline media, several noteworthy works have contributed to understanding and advancement of the concept. Previous studies have explored the application of alkaline electrochemical devices for oxygen production, emphasizing low-energy consumption and high efficiency.^30−37^ While these endeavors provided valuable insights, all the experimental setups in the mentioned studies used high concentrations of KOH solution as a liquid electrolyte, which rendered the device far from practical application by necessitating an additional process to further purify the oxygen from the electrolyte. Furthermore, the utilization of liquid KOH solutions entails potential risks, given their corrosive nature and the possibility of causing severe burns upon contact with the skin or mucous membranes. To the best of our knowledge, only three peer-reviewed studies reported the separation of oxygen from O_2_/N_2_ gaseous mixtures using AEMs over the past decade.^38−40^ Two of these studies utilized liquid KOH as the anolyte, and the third study used Argon as a sweep gas for the anode and obtained a low current density of only 30 mA cm^–2^.

**Figure 1:**
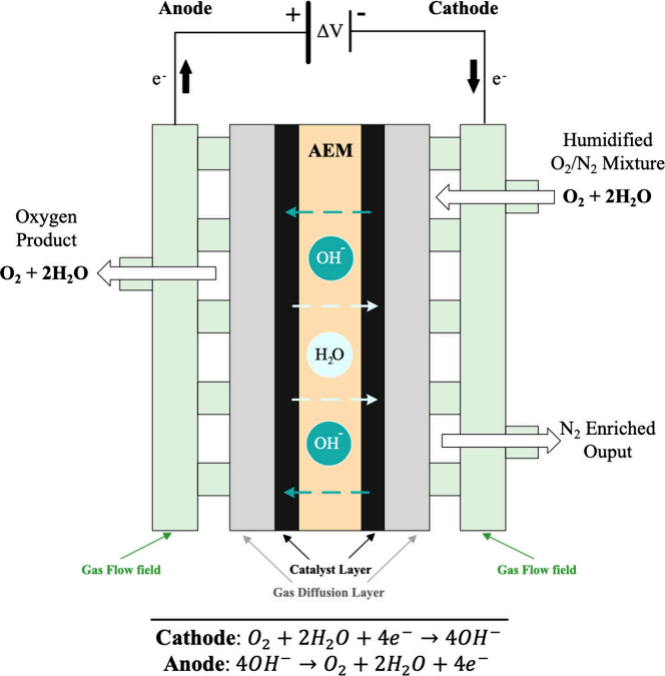
Scheme of the Anion Exchange Membrane Oxygen Separator (AEMOS).

We herein report a novel solid-state device that we call the Anion-Exchange Membrane Oxygen Separator (AEMOS). This device can extract oxygen from a mixture of oxygen and nitrogen, similar in composition to air, under a low external potential gradient using a solid polymer AEM as its sole electrolyte. To the best of our understanding, this is the first all-solid electrochemical device that can operate at low temperatures, free of liquid electrolytes or sweep gases, producing oxygen of over 96% purity. The AEMOS device is described in the schematic diagram in Figure 1.

The AEMOS electrochemical cell is comprised of an AEM between a cathode, where oxygen reduction reaction (ORR) occurs (eq. 1), and an anode, where oxygen evolution reaction (OER) occurs (eq. 2). During the operation, the cathode is fed with a humidified mixture of 79% nitrogen and 21% oxygen (synthetic air gas), and a low potential difference is applied between the cathode and anode electrodes. The OH^–^ anions generated in the cathode during the ORR are transported through the AEM to the anode, wherein oxygen, water, and electrons are produced. The total process is, therefore, a net transport of oxygen from the cathode gas mixture to the pure oxygen gas output in the anode.
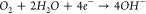
1
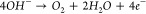
2In this study, our focus has been on pioneering a novel approach to oxygen separation from synthetic air, avoiding the use of liquid electrolytes or sweep gases in either electrode. Furthermore, we have explored the use of earth-abundant metals like Ni and Fe as electrocatalysts for the anode to develop a sustainable, green, and affordable AEMOS cell and process. Our findings demonstrate the successful concentration of oxygen from a diluted-oxygen feed stream, by using a low potential difference and low temperature. Under these unprecedented conditions, this study marks a pioneering landmark in the field of oxygen separation and oxygen generation technologies.

## 2 Methods

### 2.1 Materials

Pt/C (40% Pt on carbon black, HiSPEC 4000) was purchased from Alfa Aesar; IrO_2_ anode (catalyst loading: 1.5 mg cm^–2^ on Sigracet 35 BC) and NiFe_2_O_4_ anode (catalyst loading: 2.0 mg cm^–2^ on 316L sintered stainless steel fiber felt) were from Dioxide Materials. Toray Paper 060-TGP-H-060 with a 5% wet-proofing gas diffusion layer (GDL) and Teflon gaskets were purchased from Fuel Cell Store. Fumion anion-exchange ionomer was supplied by Fumatech BWT GmbH (Germany); AemionTM and Aemion^+TM^ AEMs were purchased from Ionomr Innovations Inc.

### 2.2 Instrumentation

Electrochemical measurements were performed using a two-electrode setup with an Ivium Vertex.S potentiostat (10A). Gas chromatography of the output gases was performed using an Agilent 7890A.

### 2.3 AEMOS Cell Fabrication and Testing

The catalyst was combined with an anion-exchange ionomer and ground with a mortar and pestle. Deionized water isopropanol (1:9 ratio) were added to the mixtures and further ground to create inks. After ultrasonication, the inks were sprayed onto the GDLs. The final catalyst loading for Pt/C cathode gas diffusion electrodes (GDEs) was 0.5 ± 0.1 mg_Pt_ cm^–2^. The membrane electrode assembly (MEA), consisting of the GDEs and AEM was assembled and conditioned in 1 M KOH. The AEMOS cells were tested in a G20 fuel cell tester (Greenlight Innovation Corp, Canada) at 60 °C and 65% relative humidity in the cathode. Polarization curves were obtained using linear sweep voltammetry from 0.0 to 1.0 V at a scan rate of 10 mV s^–1^ without *iR*-compensation.

### 2.4 Modeling Approach

A one-dimensional time-dependent isothermal model of AEMOS was developed. The computational domain, illustrated in Figure S1, comprises a five-layer MEA, consisting of cathode GDL, cathode CL, an AEM, anode GDL, and anode CL. The model accounts for mass transport across the MEA and incorporates electrochemical reactions within both CLs as well as chemical degradation of the ionomeric materials.

Full details of the methods are provided in the Supporting Information.

## 3 Results and Discussion

A triad of AEM and catalyst material combinations for the cathode and anode was used to demonstrate the adaptability and capability of the AEMOS device. We first prepared a Pt/C cathode and an IrO_2_ anode, as both of these materials are considered standard catalysts for alkaline ORR and OER, respectively, together with an Aemion^+^ 15 μm thick AEM. Two additional cells were prepared by using NiFe_2_O_4_ as the anode’s catalyst, together with Pt/C for the cathode and Aemion+ 15 μm and Aemion 25 μm AEMs.

**Figure 2:**
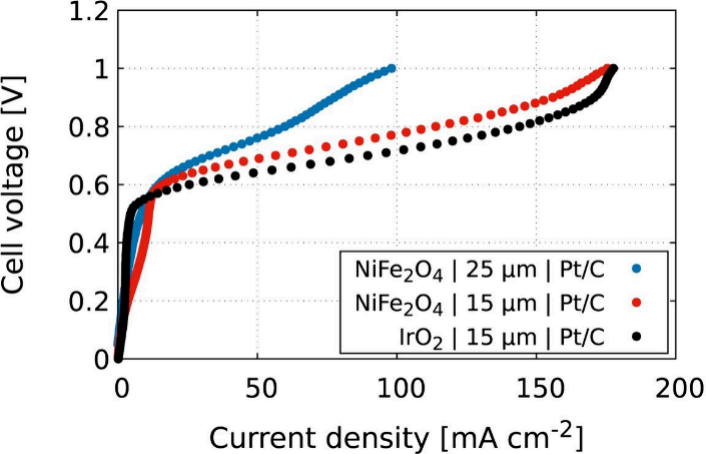
Polarization curves of AEMOS cells with various materials. Synthetic air flow to the cathode: 500 mL min^–1^, 65% relative humidity, and 60 °C.

The resultant polarization curves obtained during the operation of each cell are shown in Figure 2. High current densities of over 175 mA cm^–2^ were achieved, showing the capability of the AEMOS cell to generate oxygen. As evident from Figure 2, the voltage needed to generate the oxygen increases in a curve of similar shape to a water electrolysis polarization curve, but with a threshold voltage of ∼ 0.52–0.60 V, above which the current density starts increasing significantly. The performance of the different cells follows the order of NiFe2O4|25 μm|Pt/C < NiFe2O4|15 μm|Pt/C < IrO2|15 μm|Pt/C (where “x|y μm|z” indicates a cell consisting of x anode catalyst, AEM of thickness y, and z cathode catalyst). For instance, at 0.7 V, the NiFe_2_O_4_|25 μm|Pt/C cell achieved a current density of 33 mA cm^–2^. At the same voltage, NiFe_2_O_4_|15 μm|Pt/C achieved a higher current density of 56 mA cm^–2^. This improvement is attributed to the decreased mass transport resistance with the use of a thinner membrane (15 vs 25 μm), which is consistent with a similar phenomenon observed in AEM-based fuel cells with the use of thinner AEMs.^41^ This high current density showcases the great potential of NiFe_2_O_4_ as a precious-metal-free OER catalyst alternative for AEMOS cells, as was demonstrated in previous studies for OER water electrolyzer applications.^42,43^ The highest current density of 90 mA cm^–2^ (measured at 0.7 V) was obtained with IrO_2_|15 μm|Pt/C, marking it as the most promising candidate for further optimization and scale-up of AEMOS cells. It is worth noting that at higher voltages, higher current densities and, therefore, higher generation rates of oxygen are achieved. For instance, at 1.0 V, current densities of 175 mA cm^–2^ were obtained, showing the great potential that these novel AEMOS cells have to generate oxygen at high fluxes. Initial short-term stability test of the IrO_2_|15 μm|Pt/C cell is shown in Figure 5S.

To validate the effective separation and enrichment of oxygen, gas chromatography (GC) analyses were performed on the gaseous products obtained from the AEMOS’s anode outlet (Figure S4 and Table S5). The analyses confirmed a high oxygen concentration of 96.7 ± 1.1%, underscoring the AEMOS device’s ability to produce highly concentrated oxygen streams. The remaining 3.3% of the gaseous product’s composition is believed to be water vapor that was not dried and/or remnants of ambient air in the tubes used to collect the sample. Notwithstanding, the high purity level achieved directly from a single-stage separation represents a significant advancement compared to conventional methods such as O_2_/N_2_ separation membrane technology, where obtaining oxygen of > 96% purity would require two or three energy-intense stages.^44−46^

Table 1 shows our results and compares them with those in the literature. As can be seen, the performance of our IrO_2_|15 μm|Pt/C AEMOS cell is the highest of all reported in previous studies on related electrochemical oxygen separation systems. The superior performance of the AEMOS device could be attributed to the unique cell configuration used, where the absence of liquid electrolytes or sweep gases plays a role in reducing mass transport resistance. These results highlight the promising potential of our approach to achieve high current densities and efficient oxygen separation compared to previously reported systems.

**Table 1:** Performance Comparison of AEMOS and Previous Electrochemical O_2_ Separation Studies.

Anode	Membrane	Cathode	Electrolyte/ Sweep gas	Temperature [°C]	Current Density @ 0.7 V [mA cm^–2^]	O_2_ Flux @ 0.7 V [mL h^–1^ cm^–2^]	O_2_ purity [%]	ref
**IrO**_**2**_	**Aemion+ 15 μm**	**Pt/C**	**None**	**60**	**90**	**22.8**	**96.7**	**This work**
FeNi-LDH	Imidazole-functionalized AEM	Oxidized carbon black	1 M KOH	25	50	22.5	99.9	(38)
FeCoNi	Sustainion X37–50 grade RT	FeCoNi	1 M KOH	60	6	25.0^c^	NA^a^	(39)
Pt/C	Ni–Fe CO_3_^–2^ LDH	Pt/C	Argon	50	30^b^	5.2^c^	NA	(40)

a NA: Not available.

b Maximal current density reported, at unknown voltage.

c Maximal flux reported, at unknown voltage.

**Figure 3:**
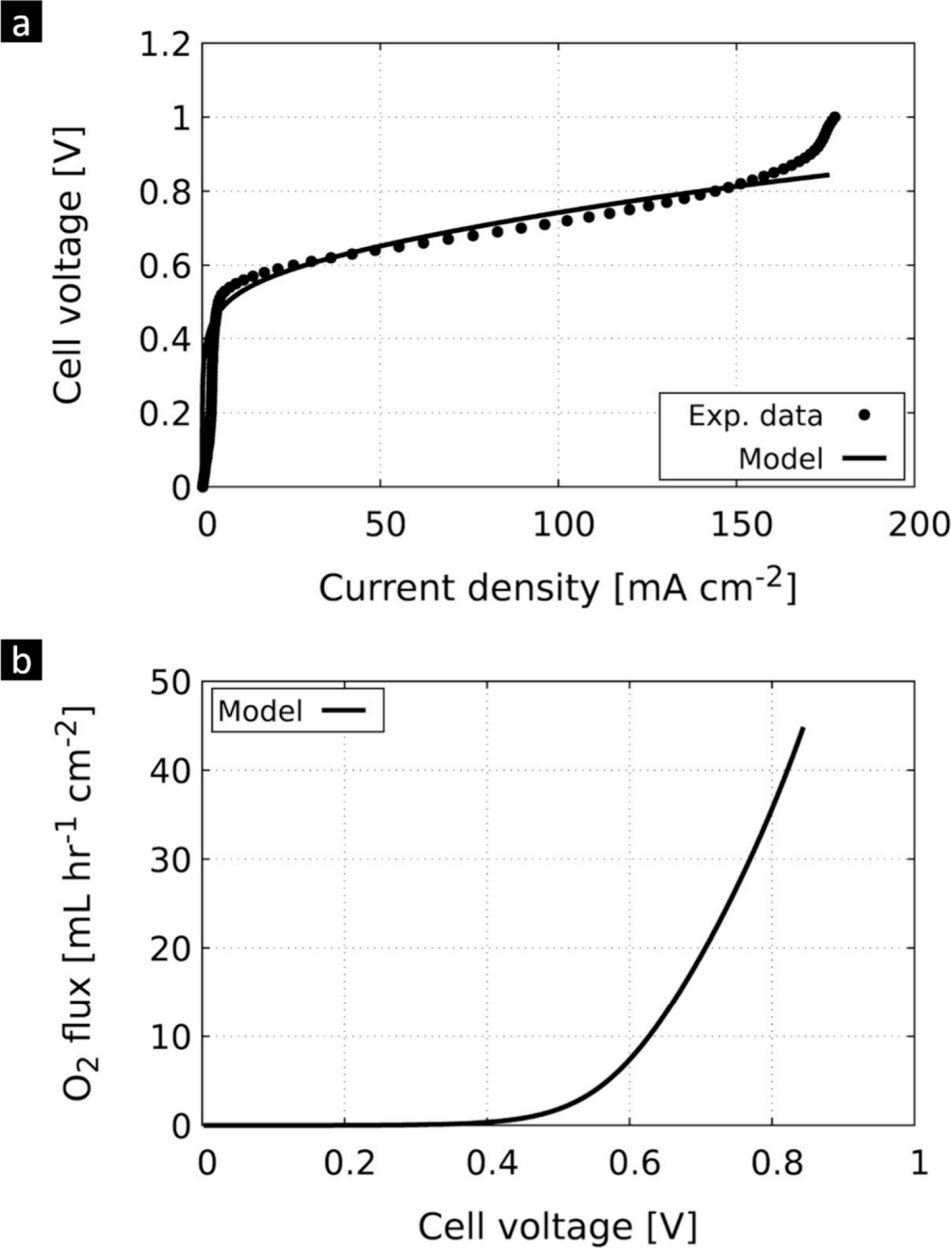
AEMOS modeling results: (a) Comparison between the simulated (solid lines) and experimental (dots) cell performance of the AEMOS IrO_2_|15 μm|Pt/C cell. (b) Calculated volumetric flux of oxygen at the anode exhaust.

Next, we employ our AEMOS-developed model to evaluate cell performance and calculate the volumetric flux of oxygen for the AEMOS cell using IrO_2_|15 μm|Pt/C. Figures 3a and 5S present a comparison between the simulated and the experimental polarization curves. The simulation results are in close agreement with the experimental data, validating the model’s ability to accurately predict the AEMOS cell performance. Some deviations from the experimental results can be seen at simulated current densities above 150 mA cm^–2^. This discrepancy between simulation and experimental results requires further investigation to understand the potential reasons. Nonetheless, these findings underscore the importance of theoretical modeling in providing insights into the behavior of AEMOS and its constituent components. Such insights can facilitate optimization efforts aimed at unlocking the full potential of AEMOS technology.

While our initial experiments yielded encouraging results, it is important to note that the reported performance of the AEMOS cells was not optimized. Further enhancement of performance is expected through systematic optimization. For instance, the type of catalyst, the structure, and the composition of the catalyst layers should be optimized. By using our developed numerical model, we can explore the theoretical capabilities of AEMOS, as showcased in Figure 3b. This figure presents the volumetric flux of oxygen vs the applied cell potential. After the threshold at ∼0.5 V, the flux of generated oxygen increases exponentially. For instance, at 0.7 V, the volumetric flux is 22.8 mL h^–1^ cm^–2^. For our 5 cm^2^ cell, the corresponding oxygen flow rate is therefore 114 mL h^–1^. In theory, when 0.7 m^2^ of an AEMOS cell is arranged in a spiral wound membrane (SWM) module with a volume of 1 L, over 2.5 L min^–1^ of oxygen can be produced. Such flow rate is sufficient for oxygen delivery to a patient via a nasal cannula or a venturi mask, as is regularly done in hospitals and intensive care unit facilities.^47^

It must be noted that our experimental setup utilized an O_2_/N_2_ mixture as a feed to prove the concept. However, this method can be used for separating oxygen from other gas mixtures. Of particular interest is the separation of oxygen from ambient air, in which case the presence of carbon dioxide may lead to the carbonation of the AEM, potentially hindering the desired reactions for oxygen separation with the formation of (bi)carbonates.^48,49^ Specifically, CO_2_ may react with hydroxide ions (OH^–^) to form carbonate ions (CO_3_^2–^) and bicarbonate ions (HCO_3_^–^). These ions may be transported through the AEM, and CO_2_ being released in the anode,^48,49^ potentially decreasing the purity of the oxygen. On the other hand, the presence of (bi)carbonate ions may increase the stability of the AEMs as the membrane polymer cations are stabilized against degradation by hydroxide in the presence of carbonate anions.^50^ Further research is therefore needed to study the AEMOS’s operation with CO_2_-containing oxygen gas mixtures.

All in all, by operating the AEMOS with different materials, we were able to successfully demonstrate this concept for the first time, showcasing AEMOS’s potential as a viable and versatile technology for future oxygen separation applications.

## 4 Conclusions

We have demonstrated a proof-of-concept for a solid-state electrochemical oxygen-separating device that effectively enriched oxygen from 21% to > 96%, with an oxygen flux of 78 mL h^–1^ cm^–2^, under low temperatures and voltages, and without the use of liquid electrolytes or sweep gases.

As a hybrid system combining principles from fuel cells (cathodic ORR) and water electrolyzers (anodic OER), this cell is unique. We developed and applied the first one-dimensional model capturing the performance of the AEMOS systems. The model successfully validated experimental data against simulated results, highlighting its potential role in future system design efforts to improve the cell performance. Our theoretical modeling highlights the prospective oxygen fluxes that can be achieved by this innovative technology; an oxygen flow rate of over 2.5 L min^–1^, suitable for venturi masks for patients, can be achieved with a compact 1 L spiral wound membrane module.

The successful extraction of high-purity oxygen from air using our AEMOS device is a significant achievement in the oxygen separation and generation fields. This innovative approach unlocks substantial potential benefits across various industries by providing a compact and energy-efficient solution for on-site oxygen enrichment. It is also expected that this novel AEMOS device can separate oxygen from different oxygen-containing gas mixtures.

## Data Availability

The data underlying this study are available in the published article and its Supporting Information.

## Supporting Information Available

Experimental procedures, modeling approach, and additional data (PDF)
