# Supplementary material for: Anion-Exchange Membrane Oxygen Separator
Source: ACS Org Inorg Au. 2024 Aug 29;4(5):498–503. doi: 10.1021/acsorginorgau.4c00052 (PMC11450738; doi:10.1021/acsorginorgau.4c00052)
Supplement: Supplementary file 1 — gg4c00052_si_001.pdf [file gg4c00052_si_001.pdf]

Supporting Information

Anion-Exchange Membrane Oxygen Separator

Maisa Faour [a], Karam Yassin [a b], Dario R. Dekel [a b c] *

[a] The Wolfson Department of Chemical Engineering, Technion – Israel Institute of Technology, Haifa, 3200003, Israel
[b] The Nancy & Stephen Grand Technion Energy Program (GTEP), Technion – Israel Institute of Technology, Haifa, 3200003, Israel
[c] The Stewart and Lynda Resnick Sustainability Center for Catalysis, Technion – Israel Institute of Technology, Haifa, 3200003, Israel

Email: Dario@technion.ac.il

Table of Contents

1. Experimental *3*

1.1. Materials 3

1.2. Instrumentation 3

1.3. AEMOS cell fabrication and testing 3

2. Modeling approach *4*

3. Gas chromatography analysis *10*

4. AEMOS performance stability *13*

References *13*

1. Experimental

1.1. Materials

Pt/C (40% Pt on carbon black, HiSPEC® 4000) catalyst was purchased from Alfa Aesar; IrO_2 anode (catalyst loading: 1.5 mg cm^{-2}, deposited on Sigracet 35 BC carbon gas diffusion layer, Ion Power) and $NiFe_2O_4$ anode (catalyst loading: 2.0 mg cm^{-2}, deposited on 316L sintered stainless steel fiber felt, Bekaert) from Dioxide Materials; (1,2) KOH (85–100.5%) and isopropyl alcohol from Biolab, Israel. Toray Paper 060-TGP-H-060 with 5% wet-proofing gas diffusion layer (GDL) and Teflon gaskets were purchased from Fuel Cell Store. Fumion® anion-exchange ionomer was supplied by Fumatech BWT GmbH (Germany); Aemion™ and Aemion+™ AEMs (AF1-HNN8-25X 25 µm thickness, and AF2-HLE8-15-X 15 µm thickness (3)) were purchased from Ionomr Innovations Inc. Drying tubes were purchased from Drierite®. Synthetic air gas (21% O_2, 79% N_2) and high-purity N_2 gas (99.999%) from Maxima, Israel, and high-purity deionized water (18.2 MΩ-cm) were used in the Anion-Exchange Membrane Oxygen Separator (AEMOS) tests.

1.2. Instrumentation

All electrochemical tests were carried out in a 2-electrode electrochemical setup using Ivium Vertex.S potentiostat (10A), purchased from BioAnalytics, Israel. Gas chromatography of the output gases was performed using Agilent 7890A Gas Chromatograph Thermal Conductivity Detector (TCD) equipped with Mole Sieve 5 Å 80/100 mesh column, according to a procedure described elsewhere. (4)

1.3. AEMOS cell fabrication and testing

Cathode gas diffusion electrodes (GDEs) were prepared for AEMOS testing following the general procedures described elsewhere. (5,6) In brief, the catalyst was combined with an anion-exchange ionomer and ground with a mortar and pestle. One part of deionized water and nine parts of isopropanol were added to the mixtures and further ground to create inks. GDLs were cut for the cathodes to active areas of 5 cm^2. After ultra-sonicating the inks at 180 W, 37 kHz for 1 h in Elmasonic P 60 H ultrasonic bath filled with water and ice to keep the temperature below 10 °C, they were sprayed directly onto the GDLs with an Iwata HP-TH airbrush. The final catalyst loading for Pt/C cathodes was 0.5 ± 0.1 mg_{Pt} cm^{-2}.

Both electrodes, along with a 9 cm^2 square piece of the AEM, were immersed in a 1 M KOH aqueous solution for 1 h, with solution changes every 20 min, to convert the membrane into its hydroxide form. The thickness of each component of the membrane-electrode assembly (MEA) was measured to ensure that proper gaskets were selected to achieve an average compression of 25%. The MEA was then assembled between Teflon gaskets, a pair of graphite plates with 5 cm^2 single serpentine gas flow fields, and two gold-plated copper current collector plates. The bolts of the cell's hardware were tightened with a torque of 2.5-3.0 Nm.

The AEMOS cells were tested in a G20 fuel cell tester (Greenlight Innovation Corp, Canada). The anode's inlet was sealed shut and the cell was heated up while flowing N_2 at 500 mL min^{-1} to the cathode and allowed to stabilize at a temperature of 60 °C. Leak tests in the cell were conducted at the beginning and end of each test to ensure good cell sealing. Once the integrity of the MEA was validated, the cathode was fed with synthetic air of 65% relative humidity (RH) at a flow rate of 500 mL min^{-1}, and polarization curves were obtained using linear sweep voltammetry from 0.0 V to 1.0 V at a scan rate of 10 mV sec^{-1} without iR-compensation. The cell voltage was then set to 0.7 V for 1-2 h, during which a gas sample from the anode outlet was dried in-line using a drying tube, collected, and analyzed in a gas chromatograph.

2. Modeling approach

We developed a one-dimensional time-dependent and isothermal model of AEMOS, expanding upon our well-established framework of the anion exchange membrane fuel cell (AEMFC) model. (7,8) We modified and updated our model to accurately capture the unique operational dynamics of AEMOS. A key distinction between the AEMFC model and our current model lies in the electrochemical reaction within the anode catalyst layer (CL). While in the cathode CL the oxygen reduction reaction (ORR) still occurs, in the anode CL the oxygen evolution reaction (OER) takes place to produce oxygen gas. The computational domain, illustrated in Figure S1, comprises a five-layer MEA, consisting of cathode GDL, cathode CL, an AEM, anode GDL, and anode CL. The model accounts for mass transport across the MEA and incorporates electrochemical reactions within both CLs.

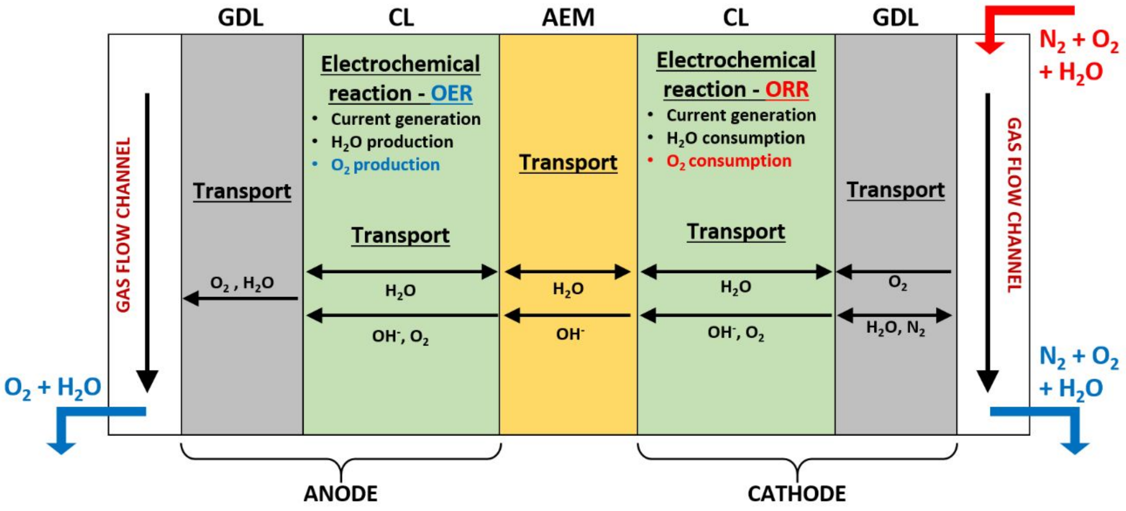

Figure S1. Overview of processes considered in the 1D model of the AEMOS cell.

In our model, the primary dependent variables include the molar concentrations of oxygen, nitrogen, and water, as well as molar velocity, partial pressure, and electric field. Water is considered in three forms: liquid, vapor, and within the ionomer. The presence of liquid water in the CL impacts the gas transport porosity. We model water transport through the membrane as a

combination of diffusion and electro-osmotic drag, while transport through the GDL is single-phase and modeled using a Maxwell-Stefan approach with Darcy's law. Electrochemical reactions are modeled using Butler-Volmer kinetics to capture the kinetics of both the OER in the anode and the ORR in the cathode.

Our numerical approach, primarily based on explicit finite differences, is described in more detail in references (7,8). The time-dependent equations are solved using an explicit scheme, while the elliptic equation for electrolyte potential is solved using the Newton-Raphson method. The algebraic equation for electrode potentials is solved using the false position method. Transport of water between the various phases in the CLs is modeled using a two-step approach: first, the amount of water in the gas and ionomer phases is updated using discretized versions of the equations of change of gas phase species concentrations, and then evaporation and absorption terms are applied, with their values calculated to satisfy local equilibrium. Finally, the electrochemical reaction term is integrated using a first-order Euler integration method.

Detailed formulation of the modified model, including the governing equations and numerical methodologies, primarily based on explicit finite differences, closely follows the approach described in the original study (8). The main model equations are presented in Table S1, with boundary conditions summarized in Table S2. Material-specific parameters and properties applied in our calculations are provided in Table S3. (8) Note that the nomenclature remains consistent with our previous studies on the AEMFC model. (7,8) By considering all of these factors, our model provides a comprehensive understanding of the behavior of the MEA and can be used to better understand and design the AEMOS cell.

Table S1. Main Model equations

**Equation/ Expression**	**Description**	**Comments**
**Transport in GDLs**		
$-\frac{c_t^g}{\varepsilon_{GDL}}\frac{\partial y_i}{\partial z} = \sum_{j,i\neq j}\frac{y_j J_i^g - y_i J_j^g}{\tau^{-2} D_{ij}^g}$	Relation between fluxes and gradients of gas phase species in the GDLs	Maxwell-Stefan approach
$v = -\frac{B_0 R}{\mu}\frac{\partial}{\partial z}\left(T c_t^g\right),\ B_0 = \frac{d_p^2}{32}$	Relation between gas phase (mass averaged) velocity and total concentration in GDL	Darcy's law
$\frac{\partial c_i^g}{\partial t} = -\frac{\partial(\varepsilon_{GDL} J_i^g)}{\partial z} - \frac{\partial\left(c_i^g v\right)}{\partial z}$	Change of gas phase species concentrations in GDLs	Convection-diffusion equation
$\sum_i Mw_i J_i^g = 0$	Relation between diffusive molar fluxes of all gas phase species	
**Transport in the CLs and the membrane**		

$-\frac{\partial}{\partial z}\left(\frac{c_i^g}{\varepsilon_g}\right) = \sum_{j,j\neq i} \frac{y_j N_i^g - y_i N_j^g}{\tau^{-2} D_{ij}^g} + \frac{N_i^g}{\tau^{-2} D_{iM}^g}$	Relation between fluxes and gradients of gas phase species in the anode and cathode CLs	Dusty gas approach
$\frac{\partial c_i^g}{\partial t} = -\frac{\partial}{\partial z}\left(\varepsilon_g N_i^g\right) + (\delta_{iO} + \delta_{iH}) R_i + \delta_{iw} E_w^g$	Change of gas phase species concentrations in anode and cathode CLs	Diffusion, mass generation due to electrochemical reactions and water evaporation
$N_w^p = -\overline{D_w}\frac{\partial}{\partial z}\frac{c_w}{\varepsilon^p} - \frac{\Theta_e J_e}{F}, \quad \varepsilon^p = \frac{c_{OH^-}^p}{IEC_m},$	Relation between fluxes and gradients of water absorbed within the ionomer	Combination of diffusion and electro-osmotic drag
$\frac{\partial c_w^l}{\partial t} = \frac{\partial}{\partial z}\left[\varepsilon^p D_w^l \frac{\partial\left(c_w^l/\varepsilon^p\right)}{\partial z}\right] + A_w - E_w + R_w^p$	Change of free water concentration in CLs	Mass generation, loss of water via evaporation, and gain of water via absorption
$0 = \frac{\partial}{\partial z} \cdot \left(\sigma \frac{\partial}{\partial z}\phi - \Theta_w F N_w^p\right) + j$	Electrolyte potential equation in membrane as well as in anode and cathode CLs	Combination of Ohm's law and diffusive potential
**Electrochemical kinetics**		
$j = j_0 f(c_i) a_v \left\{ exp\left[\frac{(1-\beta)nF\eta}{RT}\right] - exp\left[-\right.\right.$	Butler-Volmer kinetics	Oxygen evolution reaction in the anode and oxygen reduction reaction in the cathode
$\int_{anode} \varepsilon_p R_{OH^-}^p dz + \int_{cathode} \varepsilon_p R_{OH^-}^p dz = 0$	Overall conservation of hydroxide ions	All the hydroxide ions produced at the cathode are consumed at the anode
$R_i = -\frac{j}{F}\frac{\upsilon_i}{\upsilon_{e^-}}$	Rates of generation of ions in anode and cathode CLs	
**Diffusion coefficients**		

$D_{OH^-}(t) = D_{OH^-}(0) \cdot \frac{c_{OH^-}(t)^2}{c_{OH^-(0)}^2} \cdot Exp\{1.3$ [illegible] $- c_{OH^-}(0)]\}$	Local hydroxide ion diffusion in ionomer as a function of IEC (equal to c_{OH^-}), changes with time	
$\sigma(c_{OH^-},\lambda) = \frac{F^2}{RT} Q_m \left(\frac{\frac{\lambda}{1+\lambda}}{D_{w\,OH^-}} + \frac{1}{D_{OH^-\,M} \cdot \lambda} \right) \tau^{-2}$	Hydroxide conductivity in ionomer as a function of λ and IEC (equal to c_{OH^-}).	Units of hydroxide concentration – mole/liter.
$\overline{D}_w = \tau^{-2} \left(\frac{1 - x_w}{D_{wOH^-}} + \frac{1}{D_{wM}} \right)^{-1}$	Fickian diffusion coefficient of free water	Water transport in the ionomeric phase.
$D_{wM}(OH^-,\lambda,T) = 1.2 \cdot 10^{-11} \lambda x Exp\{1.3 \times 10^{-10} [c_{OH^-}(t$ [illegible] $- c_{OH^-}(0)]\} x Exp\left\{ -\frac{E_a}{R} x \left(\frac{1}{T} - \frac{1}{333} \right) \right\}$	Water diffusivity in ionomer, D_{wM}	Function of λ, T, and IEC (equal to c_{OH^-}). T = 60 °C.

Table S2. Boundary conditions

**Description**	**Expression**
GDL external boundary	$c_i^g\vert_{GDL} = c_{i0}^g$
GDL/CL boundaries (gas phase species)	$c_i^g\vert_{GDL} = c_i^g\vert_{CL}$
GDL/CL boundaries (water absorbed in ionomer)	$\left.\frac{\partial}{\partial z} c_w \right\vert_{CL} = 0$
GDL/CL boundaries (liquid (free) water)	$c_w^l\vert_{CL} = 0$

GDL/CL boundaries (electrolyte potential)	$\left.\frac{\partial}{\partial z}\phi\right\|_{CL} = 0$
CL/membrane boundaries (gas phase species)	$\left.\frac{\partial}{\partial z}c_i^g\right\|_{CL} = 0$
CL/membrane boundaries	$\lambda\|_{CL} = \lambda\|_{membrane}$

Table S3. Properties and parameters

**Parameter**		**Value**
Cell temperature		60 °C
Anode back pressure		100 kPa (absolute)
Cathode back pressure		100 kPa (absolute)
Polarization scan rate		10 mV/s
Cathode catalyst loading		0.5 mg_{Pt}/cm^2
Anode catalyst loading		2 mg_{IrO2}/cm^2
Anode	GDL thickness<br>CL thickness<br>RH	200 µm<br>20 µm<br>0%
Cathode	GDL thickness<br>CL thickness<br>RH	200 µm<br>20 µm<br>65%
Membrane thickness		15 µm
Membrane IEC		2.3 meq/g
Membrane conductivity		100 mS/cm
Maximum λ, λ_{max}		10 (Corresponding to ionomer/water contact)

Nomenclature

A_w	Volumetric rate of absorption of free water by ionomer
B_0	Geometric coefficient in Darcy law
c_i^g	Molar concentration of species "i" per unit total volume in gas phase
c_i^p	Molar concentration of species "i" per unit total volume in ionomer phase
c_w^l	Molar concentration of free (liquid) water per unit total volume
c_t	Total molar concentration per unit total volume

D_{ij}	Pair diffusion coefficient in dusty gas
D_{iM}	Effective species-dust (membrane/solid) diffusion coefficient
$\overline{D_w}$	Fickian diffusion coefficient of free water
E_w^g	Water evaporation/condensation rate
F	Faraday's constant, 96485 $C\ mol^{-1}$
j	Local electrode (volumetric) current density
J_e	Current density within ionomeric phase
J_i^g	Mass flux for a species i
j_0	Exchange current density
n	Number of electrons involved in the electrode reactions
N_i	Molar flux of species""""
P	Gas pressure
Q_m	Molar concentration of fixed positive charges in ionomer
R_i^g	Volumetric rate of production of species"""" in gas phase via electrochemical reaction
T	Temperature
Mw	Molecular weight
v	Gas flow velocity vector
y_i	Gas phase mole fraction of species""""

Greek letters

β	Charge transfer coefficient
δ_{ij}	Kronecker delta function of species"""" and""""
ε_s^g	Porosity in catalyst for gas phase
ε_{GDL}	Porosity of a GDL layer
ε_p	Effective porosity for ionomer phase
μ	Dynamic vyscosity
η	Surface overpotential
Θ_e	Electro-osmotic drag coefficient
Θ_W	Hydroxide transport coefficient in Eq. 62
λ	Water uptake (hydration number) $\frac{c_w^p + c_w^l}{c_{OH^-}^p}$
ν_i	Stochiometric coefficient of species""""
ρ^g	gas density
σ	Ionic conductivity
τ	Tortuosity
ϕ	Electric potential

3. Gas chromatography analysis

Gas chromatography analysis was employed to quantify the oxygen concentration in the gaseous products obtained from the AEMOS cells. Prior to sample analysis, a calibration curve was established by running three standard samples containing pure oxygen at varying volumes. As expected, the peak area in the chromatogram increased proportionally with the sample volume, allowing for the construction of a linear calibration plot correlating peak area to oxygen concentration.

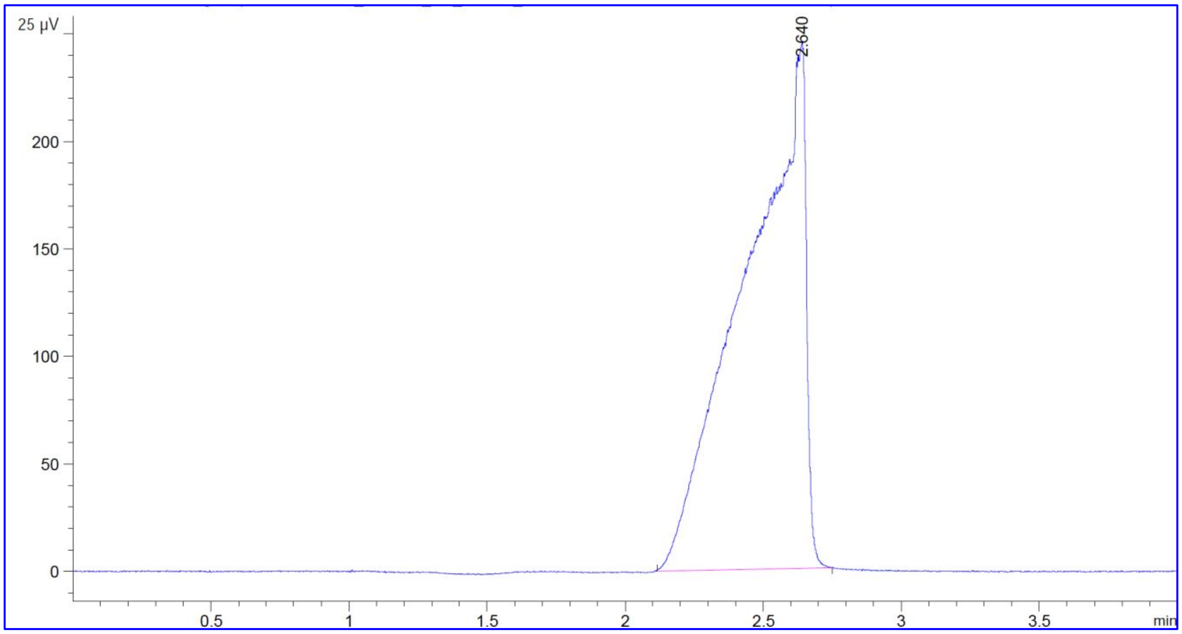

Figure S2. Gas chromatography analysis of 1.0 mL pure oxygen for calibration.

Table S4. Gas chromatography analysis parameter table for calibration.

**Volume [mL]**	**Retention time [min]**	**Peak height [25 µV]**	**Peak Area [25 µV*s]**
0.2	2.347	106.62719	783.69116
0.5	2.502	175.52016	1853.69751
1.0	2.640	242.93486	3682.53809

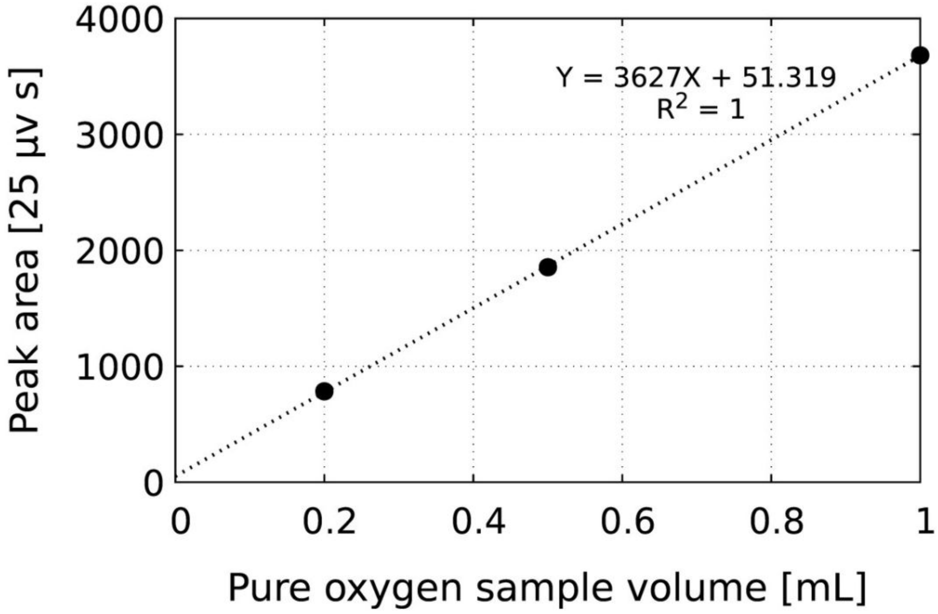

Figure S3. Calibration curve for O_2 using gas chromatography analysis peak areas of different volumes of pure O_2.

With the calibration curve in place, the gaseous sample collected from the AEMOS cell was then injected into the gas chromatograph. Three injections of 1.0 mL volume each were made from the same sample to obtain the mean and standard deviation. The area of the corresponding oxygen peak was integrated and substituted into the linear equation obtained from the calibration data. This calculation yielded the volumetric percentage of oxygen present in the sample. The high concentration of 96.6 ± 1.1% oxygen confirms the effective separation and enrichment capabilities of the AEMOS device, producing a highly pure oxygen stream directly from an O_2/N_2 mixture without the need for additional purification steps.

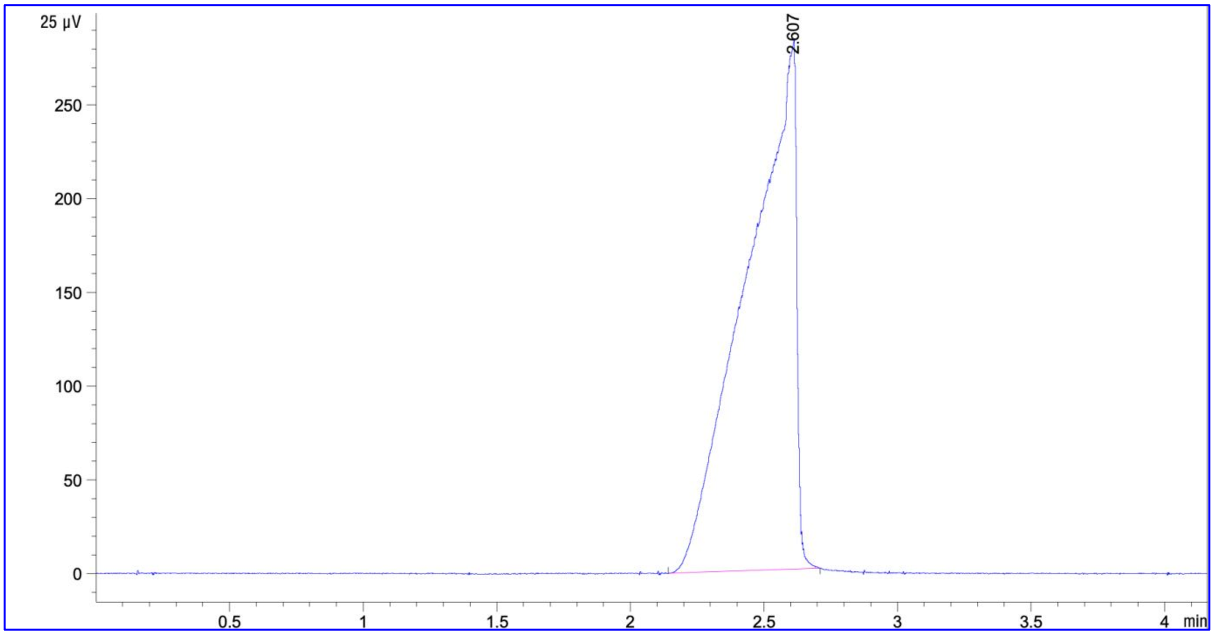

Figure S4. Gas chromatography analysis of the generated gas from the anode of the AEMOS device.

Table S5. Gas chromatography analysis parameter table for the generated gas from the anode of the AEMOS device.

Sample	Retention time [min]	Peak height [25 µV]	Peak Area [25 µV*s]	Oxygen [% v/v]
1	2.608	278.21051	3512.56250	95.42992
2	2.607	280.85574	3609.21997	98.09487
3	2.595	278.34900	3550.18750	96.46728
			**Mean:**	**96.66403**
			**Standard deviation:**	**1.09681**

4. AEMOS performance stability

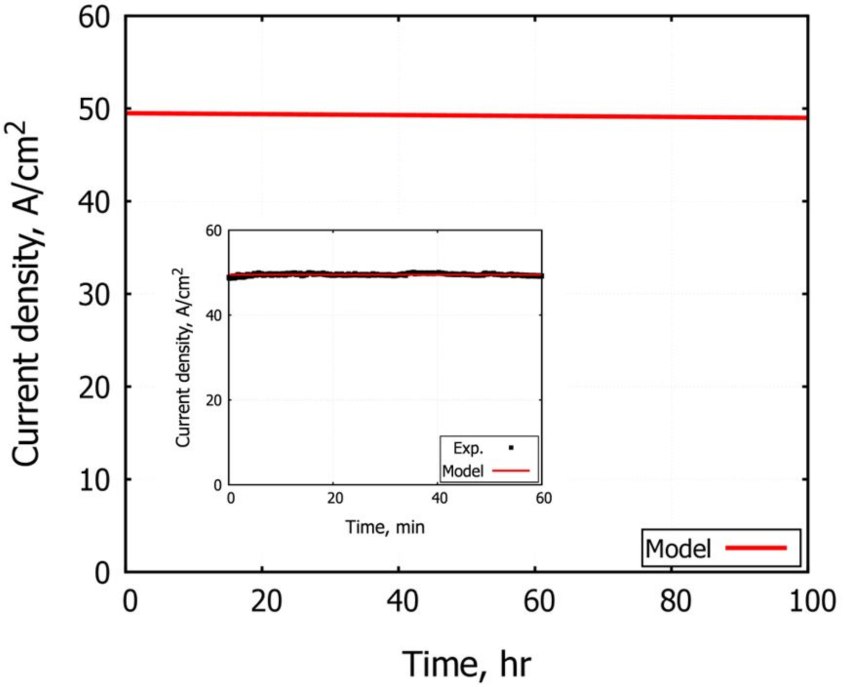

Figure S5. Simulated performance stability of AEMOS IrO_2|15 μm|Pt/C operated at a constant voltage of 0.7 V over 100 hours of operation, with a zoomed-in area showing a comparison between the experimental and model data.

References

1. Liu Z, Sajjad SD, Gao Y, Yang H, Kaczur JJ, Masel RI. The effect of membrane on an alkaline water electrolyzer. Int J Hydrogen Energy. 2017 Dec 14;42(50):29661–5.
2. Kutz RB, Chen Q, Yang H, Sajjad SD, Liu Z, Masel IR. Sustainion Imidazolium-Functionalized Polymers for Carbon Dioxide Electrolysis. Energy Technology [Internet]. 2017 Jun 1 [cited 2024 Apr 12];5(6):929–36. Available from: https://onlinelibrary.wiley.com/doi/full/10.1002/ente.201600636
3. Ionomr Innovations Inc. Aemion+® Electrolysis Offerings: Anion Exchange Membranes & Polymers Product Information. 2021 [cited 2024 May 6]; Available from: https://ionomr.com/wp-content/uploads/2021/04/FM-6028-A-Properties-of-Aemion-Water-Electrolysis-Membranes-copy.pdf
4. Zhang SX, Jiang R, Yun N, Peng R, Chai XS. A simple high-throughput headspace gas chromatographic method for the determination of dissolved oxygen in aqueous samples. J Chromatogr A. 2019 Dec 20;1608:460399.
5. Douglin JC, Sekar A, Singh RK, Chen Z, Li J, Dekel DR. Hydrogenated TiO2 Carbon Support for PtRu Anode Catalyst in High-Performance Anion-Exchange Membrane Fuel Cells. Small [Internet]. 2023 [cited 2024 Feb 21];2307497. Available from: https://onlinelibrary.wiley.com/doi/full/10.1002/smll.202307497
6. Douglin JC, Vijaya Sankar K, Biancolli ALG, Santiago EI, Tsur Y, Dekel DR. Quantifying the Resistive Losses of the Catalytic Layers in Anion-Exchange Membrane

Fuel Cells. ChemSusChem [Internet]. 2023 Dec 19 [cited 2024 Feb 21];16(24):e202301080. Available from: https://onlinelibrary.wiley.com/doi/full/10.1002/cssc.202301080
7. Dekel DR, Rasin IG, Brandon S. Predicting performance stability of anion exchange membrane fuel cells. J Power Sources. 2019 Apr 30;420:118–23.
8. Dekel DR, Rasin IG, Page M, Brandon S. Steady state and transient simulation of anion exchange membrane fuel cells. J Power Sources. 2018 Jan 31;375:191–204.
